# Movie recommendation model based on probabilistic matrix decomposition using hybrid AdaBoost integration

**DOI:** 10.7717/peerj-cs.1338

**Published:** 2023-04-21

**Authors:** Zhengjin Zhang, Qilin Wu, Yong Zhang, Li Liu

**Affiliations:** 1 Chaohu University, Hefei, China; 2 Macau University of Science and Technology, Macau, China; 3 Institute of Network and Distribution, Chaohu University, Hefei, China; 4 Sichuan film and television University, Chengdu, China

**Keywords:** Neural Network, PMF, AdaBoost, Ensemble Learning

## Abstract

In recent years, recommendation systems have already played a significant role in major streaming video platforms.The probabilistic matrix factorization (PMF) model has advantages in addressing high-dimension problems and rating data sparsity in the recommendation system. However, in practical application, PMF has poor generalization ability and low prediction accuracy. For this reason, this article proposes the Hybrid AdaBoost Ensemble Method. Firstly, we use the membership function and the cluster center selection in fuzzy clustering to calculate the scoring matrix of the user-items. Secondly, the clustering user items’ scoring matrix is trained by the neural network to improve the scoring prediction accuracy further. Finally, with the stability of the model, the AdaBoost integration method is introduced, and the score matrix is used as the base learner; then, the base learner is trained by different neural networks, and finally, the score prediction is obtained by voting results. In this article, we compare and analyze the performance of the proposed model on the MovieLens and FilmTrust datasets. In comparison with the PMF, FCM-PMF, Bagging-BP-PMF, and AdaBoost-SVM-PMF models, several experiments show that the mean absolute error of the proposed model increases by 1.24% and 0.79% compared with Bagging-BP-PMF model on two different datasets, and the root-mean-square error increases by 2.55% and 1.87% respectively. Finally, we introduce the weights of different neural network training based learners to improve the stability of the model’s score prediction, which also proves the method’s universality.

## Introduction

Today, the Internet has become an essential part of human life. Faced with the massive amount of information that appears everyday, consumers often have problems choosing too much available information. The original intention of developing a recommendation system is to help consumers effectively handle the information explosion. In recent years, recommendation systems have become widely used in streaming video platforms such as Netflix, Amazon Prime Video, Hulu, and e-commerce portals such as Amazon, eBay, and Taobao. This article focused on optimizing recommendation algorithms for streaming video platforms.

Worldwide, major video-streaming platforms are radically breaking the traditional digital entertainment habits of audiences in recent years, thus affecting or even changing the ecology of the entire film and television industry. Under the influence of the COVID-19 pandemic, audiences have increasingly reduced the frequency of watching movies in theatres and choose to enjoy movies and TV dramas at home. The rapid development of video-streaming providers and high-quality services has made increasingly audiences around the world pay for streaming services. The following Table 1 shows the statistics of the top ten video-streaming providers worldwide. These data are based on the quarterly financial reports released by major companies and the latest news online.

**Table 1:** Worldwide top 10 subscription video-streaming services.

Country	Title	Subscribers	Source date	Cost
Global	Netflix	221,600,000	2022 Q2	USD 9.99/month
Global	Amazon Prime Video	200,700,000	2022 Q1	USD 8.99/month
USA	Disney+	137,700,000	2022 Q2	USD 7.99/month
China	Tencent Video	124,000,000	2022 Q2	RMB 25/month
China	iQiyi	97,000,000	2022 Q1	RMB 25/month
China	Youku	90,000,000	2020 Q2	RMB 15/month
USA	Youtube Premium	50,000,000	2021 Q3	USD 11.99/month
USA	Hulu	45,600,000	2022 Q3	USD 5.99/month
USA	Paramount+	40,000,000	2022 Q2	USD 4.99/month
India	Eros Now	39,000,000	2021Q2	INR
				49/month

As shown in Table 1, the competition among significant streaming video platforms is booming. Whether the young but influential Disney+ and HBO Max, or Netflix, Hulu, and Amazon Prime Video have been operating for many years, the core of winning the “streaming war” is high-quality content and refined streaming services. Talking about streaming services, subscribers and providers increasingly value personalized recommendation systems. Algorithmic advantages have become an essential technical way to win in the highly competitive market. When subscribers face massive video information, the first concern is how to choose quickly. “But humans are surprisingly bad at choosing between many options, quickly getting overwhelmed and choosing ’none of the above’ or making poor choices” (Schwartz, 2004). According to Netflix’s consumer research, “A typical Netflix member loses interest after perhaps 60 to 90 s of choosing, having reviewed 10 to 20 titles on one or two screens” (Gomez-Uribe & Hunt, 2015). Compared to what the providers offer, the generally impatient audience is more concerned about where they can quickly and easily find suitable content for themselves in the massive video information. Major streaming video platforms are well aware of the importance of recommendation systems in user experience and have developed their recommendation systems. Take Netflix, the world’s largest streaming video platform and the first one to develop a movie recommendation system, as an example, “connecting people with their favorite movies” has always been the mantra for Netflix. Since 2006, starting to hold the million-dollar prize, Netflix has “developed and used our recommend system because we believe it is core to our business for some reason. Our recommend system helps us win moments of truth: when a member starts a session, and we help that member find something engaging within a few seconds, preventing abandonment of our service for an alternative entertainment option” (Gomez-Uribe & Hunt, 2015). Through continuous research and different algorithm combinations to improve the accuracy of the personalized recommendation system, Netflix helps their members quickly and accurately select their favorite videos, improving the comprehensive service experience (Lee, Xu & Lin, 2019; Abdullah, Xu & Geva, 2013).

Nowadays, consumers often subscribe to at least one paid streaming video service. Taking the United States as an example, according to relevant data researched by Deloitte Company, “At the very start of 2020, US consumers subscribed to an average of three paid streaming video services. By October, that number rose to five”.

In addition, consumer loyalty to streaming video brands is significantly lower than viewers who pay for TV; subscribers have so many choices and can easily switch video-streaming services. Beyond excellent content, focusing on the accuracy of personalized recommendation systems to retain customers could be a key. Deloitte also put forward in the survey report, “In our January 2020 survey, only 20% of respondents who subscribed to a streaming video service had cut a service in the previous 12 months, but by October, 46% had cut at least one in just the previous six months”. Therefore, optimizing the algorithm of the recommendation system is crucial for the streaming video platforms to win more subscribers and retain them (Hamacher & Buchkremer, 2022).

Recently, matrix factorization technology has developed rapidly due to its good scalability and high recommendation accuracy. Matrix factorization has received increased attention since the famous Netflix recommendation competition. The basic assumption of matrix factorization technology is that potential factors can depict the user’s preferences and project characteristics. The minimum value of the sum of squares of the distance between the original scoring matrix and the scoring matrix of potential factor characters should be determined for the best scoring matrix of the potential factor. The usual methods are probability matrix factorization, Bayesian probability matrix factorization, and fast parallel matrix factorization.

By integrating the matrix factorization model with the domain-based recommendation method, Koren, Bell & Volinsky (2009) proposed a new SVD++ model. Mnih & Salakhutdinov (2007) analyzed the principle of matrix factorization from the perspective of probability. They proposed the probabilistic matrix factorization (PMF) model (Salakhutdinov & Mnih, 2008), which extended matrix factorization to any maximum likelihood solution. Afterwards, the Bayesian Probabilistic Matrix Factorization (BPMF) was introduced (Akulwar & Pardeshi, 2017).

The idea of integrated learning has also been adopted to increase the accuracy of the recommendation system. Fang, Fu & Zhou (2011) integrated the recommendation method based on user similarity, used different similarity measures to generate different recommendation models, and weighted the sum to obtain the final prediction score, which improved the model’s prediction accuracy. Yan, Qi & Pang (2020) constructed a new dataset by combining user-based and product-based prediction score differences with actual scores and then trained the XG-boost model on the dataset. The above integration methods are all based on content-based recommendation algorithms. The algorithm will also bring disadvantages such as high time complexity and low prediction accuracy. Users or commodities with a similarity of 0 may appear when applied to high-dimensional sparse data, resulting in reduced algorithm accuracy. Marappan & Bhaskaran (2022) promoted movie recommendation system modeling using machine learning. To reduce human effort by proposing movies based on the user’s interests efficiently and effectively without wasting much time in pointless browsing, the movie recommendation system is designed to assist movie aficionados. This work focuses on developing a movie recommender system using a model that incorporates both cosine similarity and sentiment analysis. Zhu et al. (2022) proposed a novel framework named Personalized Transfer of User Preferences for Cross-domain Recommendation (PTUPCDR). With the meta-generated personalized bridge function, the user’s preference embedding in the source domain can be transformed into the target domain, and the transformed user preference embedding can be utilized as the initial embedding for the cold-start user in the target domain (Zhu et al., 2022).

Huang, Tan & Sun (2019) proposed a method “Collaborative recommendation algorithm based on probabilistic matrix factorization in probabilistic latent semantic analysis” in 2019. They promote the performance of a collaborative recommendation algorithm based on the improved probabilistic latent semantic model in this article. But they used only one evaluation metric, MAE, to measure recommendation performance. And the dataset has only Movielens, the results are relatively simple. Yang, Zheng & Zhang (2018) proposed a hybrid social network recommendation algorithm based on feature Transform and Probabilistic Matrix Factorization (TPMF). Using the Probability Matrix Factorization (PMF) method as recommendation framework, trust network, the relationship between the recommended items, user-item score matrix, and adaptive weight were combined to balance the impact of individual and social potential characteristics on users. They use the two datasets of Epinions and Ciao to validate their proposed method (Yang, Zheng & Zhang, 2018). Pujahari & Sisodia (2020) proposed a novel method “Pair-wise Preference Relation based Probabilistic Matrix Factorization for Collaborative Filtering in Recommender System”. They propose a Probabilistic MF (PMF) model that takes Preference Relation as input (instead of ratings) for generating efficient ranking of items. The user and item side information are integrated into the model using matrix co-factorization technique. The experiments use dataset-Movielens(1M) and Movielens(20M) to validate their proposed method (Pujahari & Sisodia, 2020). Shen et al. (2019) proposed a method “Sentiment based matrix factorization with reliability for recommendation” in this article. They propose the sentiment based matrix factorization with reliability (SBMF + R) algorithm to leverage reviews for prediction. They develop a sentiment analysis approach using a new star-based dictionary construction technique to obtain the sentiment score, a user reliability measure that combines user consistency and the feedback on reviews, and incorporate the ratings, reviews, and feedback into a probabilistic matrix factorization framework for prediction (Shen et al., 2019). The experiments use Amazon datasets to validate their proposed method. The proposed approach could adjust the weights of rating and sentiment information using the reliability measure.

From the analysis above, we can conclude that the probability factorization matrix has intrinsic disadvantages in dealing with high-dimensional sparsity. The study proposes a probability matrix factorization model based on the hybrid AdaBoost ensemble method. The main work includes the following: (1) The probability matrix method is used to calculate the scoring matrix of users and items with the membership function of the fuzzy matrix and the selection of the cluster centre. Compared with the traditional probability matrix method, this method’s accuracy is higher, and the scoring matrix of users and items can be better constructed. (2) The AdaBoost method in ensemble learning is put forward to build a strong learner and improve score prediction accuracy. (3) The neural network weighting mechanism is introduced, and the weights of different weak learners are trained based on the AdaBoost integration method. Finally, a strong learner is obtained, which further improves the model prediction accuracy and stability.

## Material and Methods

In this section, we review the related literature and discuss their differences from our method.

### Probabilistic matrix factorization (PMF)

**Figure 1:**
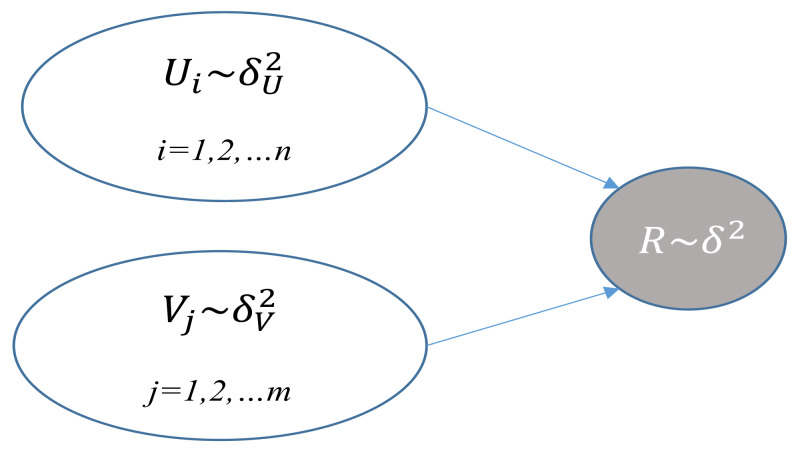
Graphical model of PMF.

PMF was put forward by Mnih & Salakhutdinov (2007). It is a famous approach for recommendation systems. Table 1 describes the notations of PMF; Fig. 1 depicts the graphical model of PMF. It is supposed that M users, N items, and a rating matrix R ∈ *R*^*k*×*N*^ and item latent matrix R ∈ *R*^*k*×*M*^ are used to reconstruct the rating matrix R. The PMF goal is to identify the optimal matrix U,V. and minimize the loss function *ɛ*, which is shown below: (refer to Table 2 for parameter description)

(1) \begin{eqnarray*}\min \nolimits \left( U,V \right) =\sum _{i}^{N}\sum _{j}^{M} \frac{{I}_{ij}}{2} { \left( {r}_{ij}-{u}_{i}^{T}{v}_{j} \right) }^{2}+ \frac{{\lambda }_{U}}{2} \sum _{i}^{N}{ \left\| {u}_{i} \right\| }^{2}+ \frac{{\lambda }_{V}}{2} \sum _{i}^{M}{ \left\| {u}_{j} \right\| }^{2}\end{eqnarray*}

**Table 2:** Notation (Zhang et al., 2021).

Notation	Description
*R*	Rating matrix
*N*	Number of users
*u*_*i*_	Latent factors of user *i*
*M*	Number of MAE
*v*_*j*_	Latent factors of item *j*
*r*_*ij*_	Rating of item *j* given by user *i*
${\hat {r}}_{ij}$	Predicted Rating of item *j* given user *i*
*U*	User latent factor
*V*	Item latent factor
*k*	Size of latent factor
*I*	Indicator, *I*_*ij*_ = 1 if *r*_*ij*_ ≠ 0,otherwise *I*_*ij*_ = 0
${\delta }^{2},{\delta }_{U}^{2},{\delta }_{V}^{2}$	Variance

After determining the objective function, the model iteratively updates U and V using the stochastic gradient descent method to minimize the function.

(2) \begin{eqnarray*}& {u}_{i}\leftarrow {u}_{i}-\alpha \cdot \left( \left( {r}_{ij}-{u}_{i}^{T}{v}_{j} \right) {v}_{j}+\lambda {u}_{i} \right) \end{eqnarray*}

(3) \begin{eqnarray*}& {v}_{j}\leftarrow {v}_{j}-\alpha \cdot \left( \left( {r}_{ij}-{u}_{i}^{T}{v}_{j} \right) {u}_{i}+\lambda {v}_{j} \right) \end{eqnarray*}

In the function, *α* is the learning rate. The iteration stops when a certain number of iterations is met or the objective function change is less than a certain threshold. Finally, the score is predicted by the trained U, V feature matrix.

### Fuzzy c-means (FCM)

Fuzzy c-means (FCM) is an unsupervised clustering algorithm. Each point has a certain strength of association between the nodes and the particular community (Katarya & Verma, 2018).

The objective function *J*_*f*_ of FCM is below:

(4) \begin{eqnarray*}{J}_{f} \left( \vec{U},\vec{C} \right) =\sum _{i=1}^{n}\sum _{j=1}^{k}{u}_{if}^{f}{ \left\| {x}_{i}-{c}_{j} \right\| }^{2}\end{eqnarray*}

where *u*_*ij*_ is the membership degree of the *i*-th node to the *j*-th cluster and ${d}_{ij}= \left\| {x}_{i}-{c}_{j} \right\| $ is the distanced between the i-th node and the center of the j-th cluster. During optimizing *J*_*f*_, the constraint ${\mathop{\sum }\nolimits }_{j=1}^{k}{u}_{ij}=1$ must be reached. The membership function is a concept of fuzzy logic, which represents the degree of belonging to a certain category. Generally, the value ranges from 0 to 1. The 0 means not belonging to a certain category, the 1 means belonging to a certain category, and the rest of the intermediate values represent the true degree of belonging to a certain category. The objective function means being as close as possible to the class center of the same class and as far as possible from the class center of a different class. The research aim is mainly to get the minimum value. As f turns out to be larger, the process is fuzzier. The *c*_*j*_ can be worked out by the equation below (Salakhutdinov & Mnih, 2008):

(5) \begin{eqnarray*}{c}_{j}= \frac{\sum _{i=1}^{n}{u}_{ij}^{f}{x}_{i}}{\sum _{i=1}^{n}{u}_{ij}^{f}} \end{eqnarray*}

The *u*_*ij*_ can be worked out by the equation below:

(6) \begin{eqnarray*}{u}_{ij}= \frac{1}{\sum _{l=1}^{k}{ \left( {d}_{ij}/{d}_{lj} \right) }^{2/(f-1)}} \end{eqnarray*}

The *J*_*f*_ can be minimized by iterative optimization with the update of membership degree *u*_*ij*_ and the cluster center *c*_*j*_ (Katarya & Verma, 2018).

### Ensemble learning

Ensemble learning means using a series of base learners for learning, and then based on a certain rule to integrate the learning results to obtain a better learning method than a single learner. Usually there are distinctions between base learners, including differing algorithms or the same algorithm with differing parameters or hyperparameters. In general, the greater the distinction between base learners, the better the final learning outcome. Ensemble learning has an obvious edge in performance improvement. That is why it is extensively used in theoretical research and practical applications. The significant ensemble learning methods are mainly Bagging and Boosting. The AdaBoost method is used in this study, so we would introduce this method’s principle in detail (Zhang et al., 2021).

AdaBoost (Ichihashi et al., 2008) is one of the most successful ensemble learning algorithms that iteratively selects several classifier instances by maintaining an adaptive weight distribution over the training examples. AdaBoost forms a linear combination 20 of selected classifier instances to create an overall ensemble. AdaBoost-based ensembles rarely over-fit a solution even if a large number of base classifiers in-stance are used (Lei & He, 2017) and it minimizes an exponential loss function by fitting a stage-wise additive model (Wu & Wu, 2013). As the minimization of classification error implies an optimization of a non-smooth, non-differentiable cost function which 25 can be best approximated by an exponential loss (Koren, Bell & Volinsky, 2009), AdaBoost therefore per-forms extremely well over a wide range of classification problems.

AdaBoost’s algorithm idea is to combine the outputs of multiple weak classifiers to produce a more efficient classification. The main steps of the algorithm are to select the weak classifier and sample dataset, select m groups of data from the dataset as the training set, and the training weight of the dataset is 1/m. Then the weak classifier is used to iterate the training T times. After each training, the training data is updated according to the training output, and a larger weight is given to the training data that failed to classify, which gives more attention to the training failure data during the next weak classifier training. A sequence of classifier functions *f*_1_, *f*_2_, *f*_3_, …, *f*_*T*_ is obtained by repeated iteration training of the weak classifier. At the same time, each classifier is given a corresponding weight, and the function with the better classification result has the greater weight. After T iterations of training, the final strong classifier is weighted by the weak classifier (Bao-an, Haijing & Jin-xiang, 2001).

**Figure 2:**
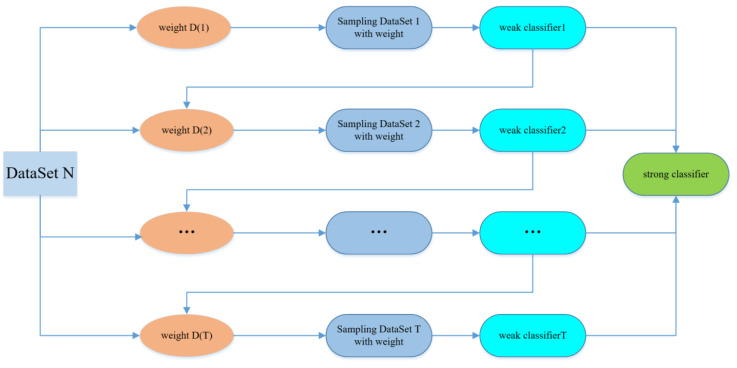
AdaBoost model.

Figure 2 indicates the structure of AdaBoost Model.

### Artificial neural network

Neural networks are composed of three parts: input layer, hidden layer and output layer, which can achieve continuous nonlinear mapping.

**Figure 3:**
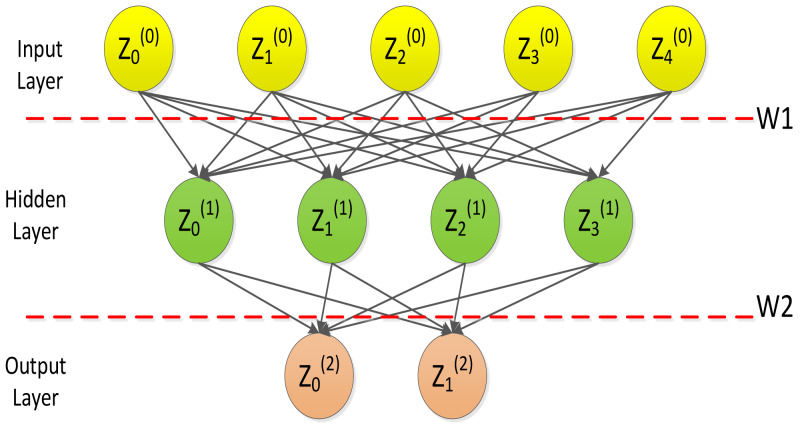
Artificial neural network model.

Neural network is a multilayer feed-forward neural network characterized by forward propagation of signals and backpropagation of errors. In the forward transmission process, the signal is processed layer by layer from the input layer, through the hidden layer, and then to the output layer. Figure 3 depicts the typical architecture of a three layer artificial neural network model (Wang, Wang & Yeung, 2015).

Each connection is associated with a numeric number called weight. The output, *h*_*i*_, of neuron i in the hidden layer is

(7) \begin{eqnarray*}{h}_{i}=\sigma \left( \sum _{j=1}^{N}{W}_{ij}{x}_{j}+{T}_{i}^{hid} \right) .\end{eqnarray*}

where *σ*() is called activation (or transfer) function, N the number of input neurons, *W*_*ij*_ the weights, *W*_*ij*_ inputs to the input neurons, and ${T}_{i}^{hid}$ the threshold terms of the hidden neurons. The purpose of the activation function is, besides introducing nonlinearity into the neural network, to bound the value of the neuron so that divergent neurons do not paralyze the neural network. A common instance of the activation function is the sigmoid (or logistic) function defined as:

(8) \begin{eqnarray*}\sigma \left( u \right) = \frac{1}{1+\exp \nolimits (-u)} \end{eqnarray*}

BP neural network is a multi-layer feed-forward neural network featured by forward propagation of signals and backpropagation of errors. In the forward transmission process, the signal is processed from the input layer through the hidden layer, and finally to the output layer.

Figure 3 indicates the basic processing framework for BP neural networks, where *Z* = (*Z*_1_, *Z*_2_, …, *Z*_*n*_) is a set of n values from an external input or output from another neuron; *W* = (*W*_1_, *W*_2_, …, *W*_*n*_) is the weight, illustrating the strength of the connection between a neuron and other neurons; ∑*WZ* is an activation value, which is equivalent to the total input of an artificial neuron; O means the output of a neuron; b is the threshold of that neuron. The weighted sum of the signs is higher than b, and the artificial neuron is activated. Accordingly, the output of artificial neurons can be depicted here:

(9) \begin{eqnarray*}O=f(\sum WZ-b)\end{eqnarray*}

In Eq. (7), f(⋅) is called an activation function. This activation function in this study is a nonlinear transformation function, which is the bipolar sigmoid -shaped function (tanh(x) function). In the procedure of error backpropagation, there exists the problem of differentiation of the activation function. The tanh(x) function can effectively address the point of derivative discontinuity and the matter of zero-centered output, that is why it is the activation function in the study. It is defined as follows:

(10) \begin{eqnarray*}f \left( x \right) =(1-{e}^{-x})/(1+{e}^{x})\end{eqnarray*}

**Table:** 

**Algorithm1:** The algorithm flow of AdaBoost- BP algorithm
**Input:** The normalized rating matrix from training data set D =$ \left\{ { \left( {x}_{i},{y}_{i} \right) }_{i=1}^{m} \right\} $,
**Output:** The rating prediction result of this sample x of this test set
1: for *t*=1,…,*k* do(*k* is the number of base models)
1.1: Randomly select cluster center with *FCM* and calculate the fuzzy membership matrix *F* with membership function. *F* matrix represents an association between the clusters for ratings of users. Select k-1 samples from the training set
1.2: Training the BP neural network on this sample to obtain the base model
2: Reduce the weight of the correct classifier and increase the weight of the weak classifier
3: The strong classifier is used to test the data set

This article uses a three-layer BP neural network with one hidden layer structure to simulate the change of the model.

### Application of probability matrix factorization model

In order to further improve the accuracy of PMF model in predicting score, the FCM method is used for score data in preprocessing data. Firstly, the FCM algorithm is suitable for solving the problems of high-dimensional and sparse data and has the advantages of strong scalability; secondly, it can solve the shortcomings of hard clustering, but express the degree of a class’s score belonging to a class in the form of membership function. Finally, this method enhances the robustness of data (Luo et al., 2014).

### Algorithm thought

Given the user’s score R, FCM uses membership degree *u*_r,j_ to represent the degree of association between user u and cluster j. Assuming that there are n user scores and k clusters, the results of fuzzy clustering satisfy the following three conditions at the same time: (1) for each user’s score r and cluster j, there exists 0 ≤ *u*_r,j_ ≤ 1. The degree to truth which the score r is attributed to the clustering center j. (2) for each user’s score r, there is ${\mathop{\sum }\nolimits }_{\mathrm{j}=1}^{\mathrm{k}}{u}_{\mathrm{r},\mathrm{j}}=1$. The score r belongs to the sum of all the different categories should be 1. (3) for each cluster j, there is $0\lt {\mathop{\sum }\nolimits }_{\mathrm{j}=1}^{\mathrm{n}}{u}_{\mathrm{r},\mathrm{j}}\lt \mathrm{n}$. For each cluster, the sum of all membership degrees in the cluster does not exceed the total number of users.

In fuzzy clustering algorithm, a membership matrix needs to be generated, and a fuzzy similarity matrix needs to be constructed for data similarity in matrix. The methods of constructing fuzzy similarity matrix include maximum and minimum calculation method, cosine angle method, and correlation coefficient method. In terms of parameter selection, the correlation coefficient method can get better clustering effect, which is generally the default method. This article mainly adopts the correlation coefficient method.

### Algorithm description

**Figure 4:**
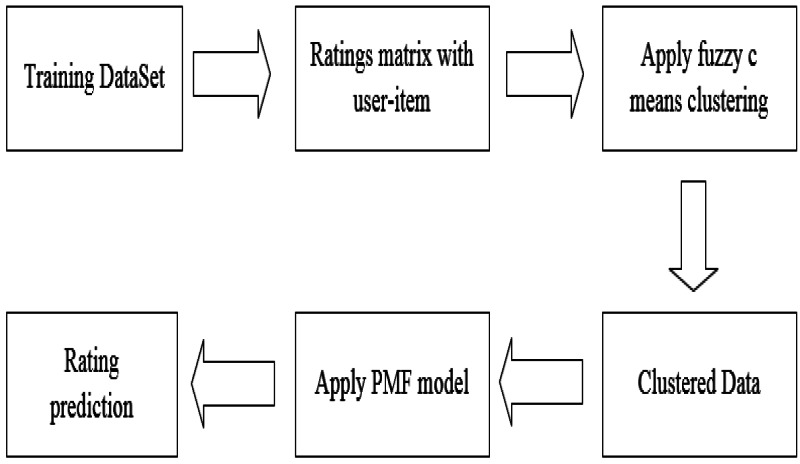
Algorithm workflow.

In Fig. 4, we show the workflow of the model. Firstly, the rating matrix is constructed from the training data, and then FCM is used to calculate the similarity and complete the clustering. Finally, the clustered data is applied to the PMF model to predict the user’s rating matrix.

**Table:** 

**Algorithm 2:** Prediction algorithm flow of PMF-FCM
**Input:** Constructing the rating matrix based on data set D =$ \left\{ { \left( {x}_{i},{y}_{i} \right) }_{i=1}^{m} \right\} $,
**Output:** Using test data to predict the result of rating matrix
1: Related parameters’ initialization
2: The clustering center is randomly selected by FCM, and the fuzzy membership matrix *F* is calculated by the membership function. The *F* matrix represents the association between user-rated clusters.
3: The clustered data is applied to the PMF model, and initialize the matrix of P and Q with gauss distribution
4: Prediction of rating matrix

### PMF model based on AdaBoost ensemble method

The efficiency and prediction accuracy of the PMF model and the recommendation algorithm based on similarity have been greatly improved. However, due to the randomness and high-dimensional sparsity of data, the model is unstable, which affects the accuracy of recommendation.

The AdaBoost ensemble learning method solves the problem of low accuracy of a single weak learning algorithm and has good generalization ability, which effectively improves the accuracy of rating prediction.

### Algorithm introduction

For the regression task, (x, y) represents a piece of data on dataset D, where x is the eigenvector and Y is the real value. We train with the multiple regression model, put the features into the regression model and produce the corresponding prediction value Φ(*x*, *D*). The predicted values on multiple data sets are integrated according to the strategy D.

(11) \begin{eqnarray*}{\Phi }_{A} \left( x \right) ={E}_{D}\Phi (x,D)\end{eqnarray*}

where x represents the input data and Y is the output value, then

(12) \begin{eqnarray*}{E}_{D}{ \left( y-\Phi (x,D) \right) }^{2}={y}^{2}-2y{E}_{D}\Phi \left( x,D \right) +{E}_{D}{\Phi }^{2} \left( x,D \right) \end{eqnarray*}

Eq. (9) and inequality $E{Z}^{2}\geq { \left( EZ \right) }^{2}$, then Eq. (10) can be changed into the following:

(13) \begin{eqnarray*}{E}_{D}{ \left( y-\Phi (x,D) \right) }^{2}\geq { \left( y-{\Phi }_{A} \left( x \right) \right) }^{2}\end{eqnarray*}

It can be seen from inequality (Eq. (11)) that the root mean square error of the predicted value ${\Phi }_{A} \left( x \right) $ generated by ensemble is smaller than the average value of root mean square error Φ(*x*, *D*), and the more unstable $\Phi \left( x,D \right) $ is, the greater the performance improvement of the ensemble method of the model.

### Algorithm description

**Figure 5:**
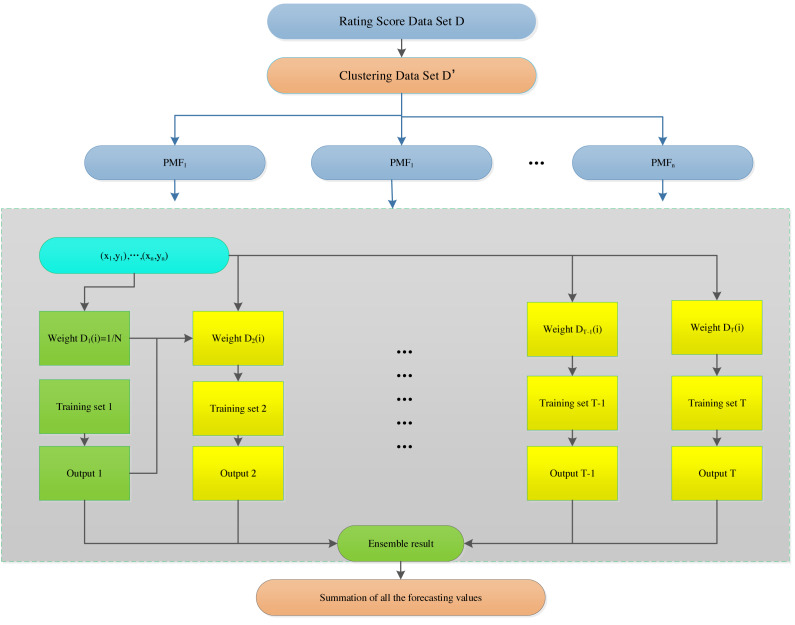
Probabilistic matrix factorization based on the hybrid AdaBoost method.

Figure 5 shows the overall structure of the model and, and the algorithm is described as follows

**Table:** 

**Algorithm3:** The algorithm flow of hybrid Adaboost method in PMF model
**Input:** Constructing the rating matrix from training data set *D*=$ \left\{ { \left( {x}_{i},{y}_{i} \right) }_{i=1}^{m} \right\} $, Initialize related parameters
**Output:** The rating prediction result of the AdaBoost ensemble learning
1:for *t* = 1,…,T do (*T* is the number of learning rounds)
1.1: Initialize the weight distribution ${D}_{t} \left( i \right) =1/m$; and train a base learner from *D* using distribution *D*_*t*_,
1.2: Each base learner with BP neural network to train the model, the weight of different base learner is adjusted according to the learner results of different the base learner.
2: Finally, and the final learner is derived by weighted majority voting of the base learners, where the weights of the learners are determined during the training process.
3: The final learner is used to test the data set.
4: The clustered data is applied to the PMF model, and initialize the matrix of P and Q with Gauss distribution.
5:Rating prediction

## Experiments

In this part, we mainly test our hypothesis through several groups of experiments: FCM method is used in PMF model to improve the accuracy of score prediction. The standards that we use are mean absolute error (MAE) and root mean square error (RMSE).

(14) \begin{eqnarray*}MAE= \frac{\sum _{{r}_{ij\epsilon C}} \left\vert {\hat {r}}_{ij}-{r}_{ij} \right\vert }{N} \end{eqnarray*}

(15) \begin{eqnarray*}RMSE=\sqrt{ \frac{\sum _{{r}_{ij\epsilon C}}{ \left\vert {\hat {r}}_{ij}-{r}_{ij} \right\vert }^{2}}{N} }\end{eqnarray*}

Where ${\hat {r}}_{ij}$ is the prediction of rating matrix, *r*_*ij*_ is the actual prediction of the rating matrix, and N is the number of data sets. According to the definitions of MAE and RMSE, MAE can better reflect the error, while RMSE is more sensitive to outliers. The mean square root of the sum of the squared error between the predicted ratings and the actual ratings is calculated to predict the accuracy. The smaller the RMSE value, the better the recommendation quality. The smaller the values of both of MAE and RMSE, the higher the accuracy of recommendation.

### Relevant parameter settings

In this experiment, we take 80% of the data as training data and 20% as test data. The relevant parameters in the experiment are set to *λ*_*U*_ = *λ*_*V*_ = *λ*_*bu*_ = *λ*_*bi*_ = 0.01, the learning rate of SGD is *α* = 0.03. The number of hidden layers of BP is 100. The experimental data sets are Movielens and FilmTrust, which are respectively applied to PMF, FCM-PMF, AdaBoost-SVM-PMF and AdaBoost-BP-PMF models for comparison and conclusion.

This experiment is carried out on Movielens and FilmTrust datasets, both of which contain the user’s rating information of the project. Both of the data sets are high-dimensional sparse matrices, with score values of 1-5 discrete values, and sparsity of 4.47% and 1.04% respectively. Table 3 as below shows the specific information of two data sets:

**Table 3:** Two datasets information (Zhang et al., 2021).

Dataset	Number of Users	Number of Items	Number of scoring records	Sparsity
Movielens	6,450	3,706	1,000,209	4.47%
FilmTrust	1,642	2,071	35,497	1.04%

### Experiment

To verify the actual recommendation accuracy effect of the model, the PMF model based on hybrid AdaBoost ensemble method is evaluated by experiments, and the results are compared with the models PMF, FCM-PMF, Bagging-BP-PMF, AdaBoost-SVM-PMF in two datasets. Table 4 shows that the comparison results of the different models in the two datasets as below:

**Table 4:** Comparison result of RMSE and MAE in Movielens.

Model	RMSE	MAE
PMF	0.93052	0.75467
FCM-PMF	0.83781	0.74131
FCM-Bagging-BP-PMF	0.79765	0.73074
AdaBoost-SVM-PMF	0.85799	0.74452
AdaBoost-BP-PMF	0.77215	0.71836

**Figure 6:**
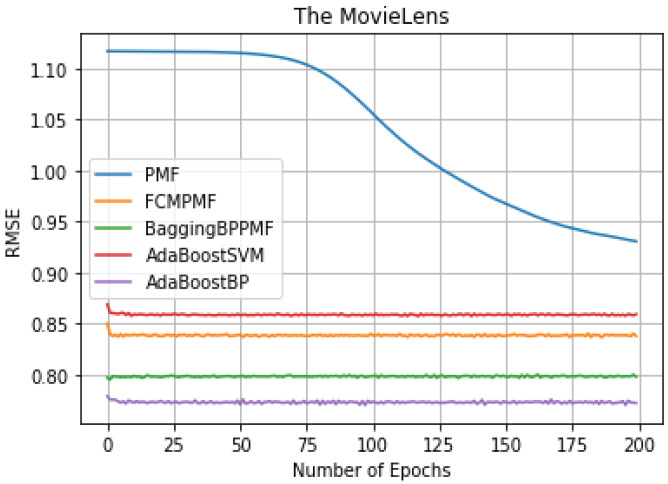
RMSE comparison result.

**Figure 7:**
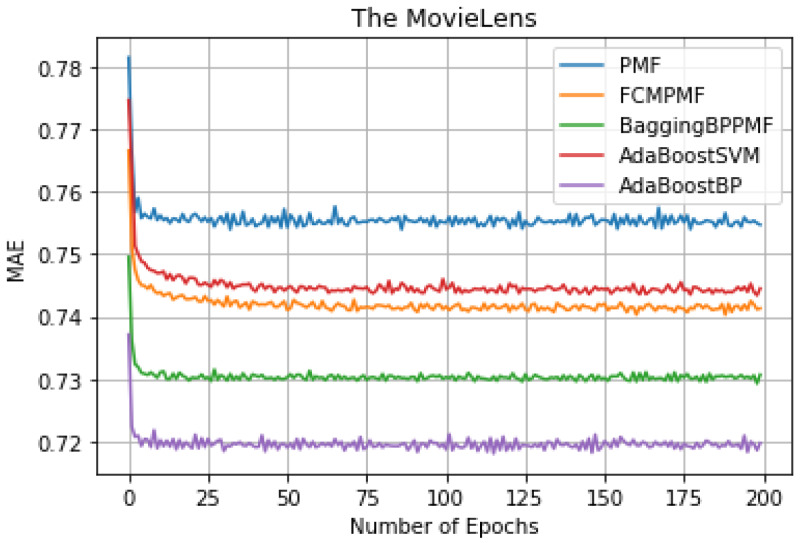
MAE comparison result.

It can be seen from Table 3, we can conclude that the performance of AdaBoost method in these models is better than the bagging method. The RMSE and MAE of the FCM-Bagging-BP-PMF model and the PMF model are about 0.79765 and 0.73704, respectively. The RMSE and MAE of the AdaBoost-BP-PMF model and the AdaBoost-BP-PMF model are about 0.77215 and 0.71836, respectively, which were improved by 2.55% and 1.87%. Compare with the performance of RMSE and MAE, AdaBoost- SVM-PMF is only better than the PMF model, lower than the other models. Finally, compared with the FCM-PMF model, the RMSE and MAE of the PMF model based on the hybrid AdaBoost method were improved by 6.495% and 2.295%. The results of the five models in RMSE and MAE are shown in Figs. 6 and 7.

**Figure 8:**
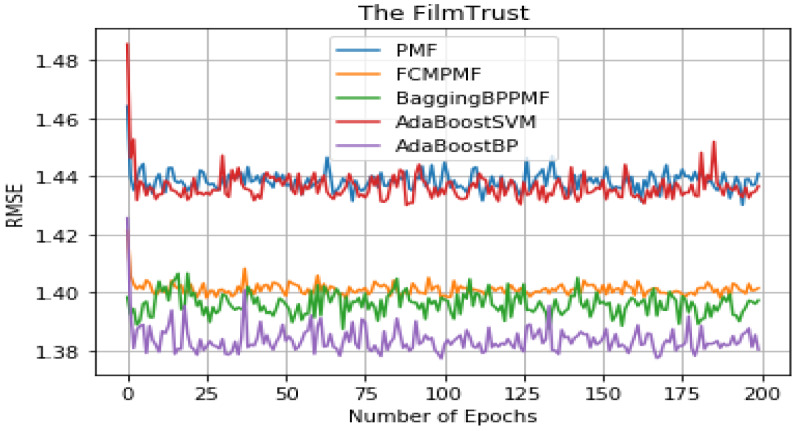
RMSE comparison result.

**Figure 9:**
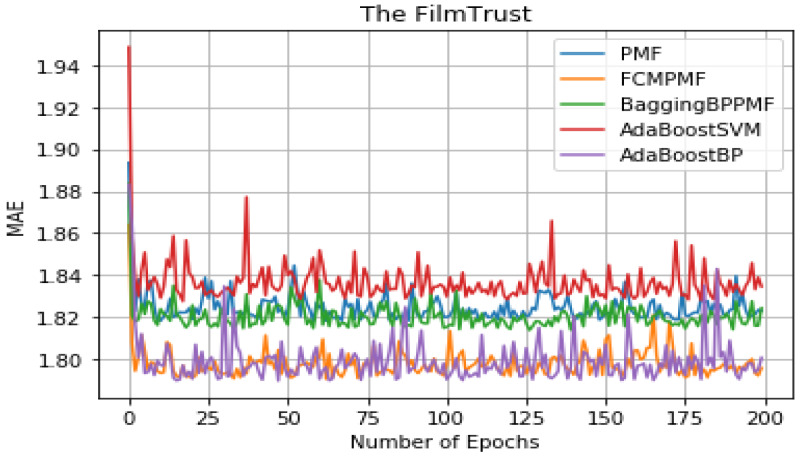
MAE comparison result.

It can be seen from Table 5 that the overall performance of the five models. The RMSE and MAE of the AdaBoost method is better than the other models’ method and they are about 1.37855 and 1.79514, respectively. The RMSE and MAE of FCM-Bagging-BP-PMF model method are about 1.39723 and 1.79593, respectively. Compared with the FCM-Bagging-BP-PMF method, the MAE is similar, but RMSE is improved by 1.1.87%. Finally, compared with the FCM-PMF model, the RMSE and MAE of the PMF model based on hybrid AdaBoost method were improved by 2.56% and 2.8%. The comparison results of the five models in RMSE and MAE are shown in Figs. 8 and 9.

**Table 5:** Comparison result of RMSE and MAE in FilmTrust.

Model	RMSE	MAE
PMF	1.44094	1.83424
FCM-PMF	1.40143	1.82315
FCM-Bagging-BP-PMF	1.39723	1.79593
AdaBoost-SVM-PMF	1.43664	1.82923
AdaBoost-BP-PMF	1.37855	1.79514

## Conclusions

Streaming media platforms occupy an increasingly important position in people’s entertainment life. People are facing an increasingly difficult choice of digital entertainment. We believe that the continuous improvement of the accuracy of the recommendation system will continue to play an important role and increasingly highlight its commercial value. Optimized algorithms can effectively guide people to quickly make their own choices and improve their experience of digital film and television entertainment consumption. For streaming media providers, the progress of algorithm accuracy can be transformed into the commercial value of the platforms, attracting more members and retaining them.

This article proposes that the PMF model is based on the hybrid AdaBoost method. FCM is used to calculate the similarity of the rating matrix of the user-item, which effectively solves the improvement of rating accuracy. Each weak learner uses BP neural network to find the optimal weight and then carries out integrated processing to construct a strong learner. The PMF model is built on a solid classifier to improve the stability of the model prediction rating matrix.

##  Supplemental Information

10.7717/peerj-cs.1338/supp-1 Supplemental Information 1 The public data sets of Movielens (1M) and FilmTrust

10.7717/peerj-cs.1338/supp-2 Supplemental Information 2 Code (runtime for Python)

10.7717/peerj-cs.1338/supp-3 Supplemental Information 3 Dataset Source Description

## Additional Information and Declarations

### Data Availability

The following information was supplied regarding data availability: The code and datasets are available in the Supplementary Files. The original sources for the datasets are: - ml-100k and ml-1m: http://www.grouplens.org/. - filmtrust: https://guoguibing.github.io/librec/datasets.html.
