# Supplementary material for: Movie recommendation model based on probabilistic matrix decomposition using hybrid AdaBoost integration
Source: PeerJ Comput Sci. 2023 Apr 21;9:e1338. doi: 10.7717/peerj-cs.1338 (PMC10280431; doi:10.7717/peerj-cs.1338)
Supplement: Supplemental Information 3 [file peerj-cs-09-1338-s003.docx]

Dataset Source Description

|  | Dataset | ORIGINAL SOURCE | Description |
| --- | --- | --- | --- |
| 1 | ml-100k | <http://www.grouplens.org/> | Public dataset |
| 2 | ml-1m | <http://www.grouplens.org/> | Public dataset |
| 3 | filmtrust | https://guoguibing.github.io/librec/datasets.html | Public dataset |
